# Port-site tumour recurrence of oral squamous carcinoma following percutaneous endoscopic gastrostomy: a lesson to be aware of

**DOI:** 10.1186/1477-7819-4-64

**Published:** 2006-09-19

**Authors:** Ian R Daniels

**Affiliations:** 1 Pelican Cancer Foundation, North Hampshire Hospital, Aldermaston Road Basingstoke Hampshire RG24 9NA, UK

## Abstract

**Background:**

Patients with aero-digestive malignancy will often require a feeding gastrostomy during their treatment to maintain their nutritional status. These are usually placed percutaneously using an endoscopic technique.

**Case presentation:**

A fifty-six year old male underwent placement of a percutaneous gastrostomy (PEG) prior to commencement of his treatment for an oral squamous cell carcinoma. The treatment for this was locally curative. However, he developed a metastasis at the site of his PEG tube. This was excised *en-bloc *with the anterior gastric and abdominal walls.

**Conclusion:**

Tumour implantation into wounds has been previously reported. In this case the direct trauma of passing the PEG tube through the oropharynx led to implantation of cells in the anterior abdominal wall. In these cases laparoscopic placement may be more beneficial to avoid this problem.

## Background

Percutaneous endoscopic gastrostomy (PEG) is commonly used in patients with malignancy of the head and neck to allow enteral nutrition to continue during the peri-operative period. We report a case of a patient who underwent PEG placement prior to surgery and adjuvant radiotherapy for a squamous carcinoma of the tongue. Six months post-resection the patient re-presented with a recurrent tumour at the PEG site.

## Case-presentation

**Figure 1:**
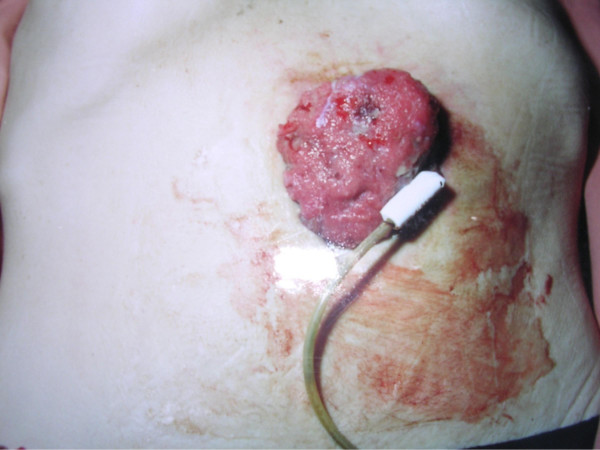
The patient's PEG site at presentation

The patient, a fifty-six year old man, presented with a squamous cell carcinoma of the floor of the mouth with metastases to the cervical nodes. He underwent PEG insertion prior to radical neck dissection with a pectoralis major flap reconstruction. Following surgery he underwent a six-week course of adjuvant radiotherapy. The patient represented three-months later with an iron-deficiency anaemia and granulation tissue around the PEG site. He was fully investigated with ultrasound scanning, endoscopy, and computerized tomography. No cause for his anaemia was found. Three months later he again presented with iron-deficiency anaemia and the area around the PEG site had grown in size (Figure 1). This was biopsied and proved to be a squamous carcinoma. He was then referred to this unit for consideration for surgery. He was not initially keen on surgery and after discussion underwent a trial of chemotherapy which after eight weeks had made no difference to the size of the tumour. He then accepted the need for surgery. At operation the tumour mass, anterior abdominal wall and anterior gastric wall were excised en-bloc with the PEG tube. A feeding jejunostomy tube was inserted at the time of operation. Histology showed squamous carcinoma identical to the original tumour with complete excision.

## Discussion

Patients with malignancy of the head and neck are often malnourished secondary to odynophagia or oropharyngeal obstruction. Because of the nature of the surgery, swallowing may be impaired post-operatively and the patient's nutritional status worsens. As the remainder of the gastrointestinal tract remains functional, enteral feeding is the favoured route of nutritional support. Nasogastric feeding is contraindicated because of gastrointestinal reflux, aspiration, nasal ulceration and frequent tube blockage [1].

Percutaneous endoscopic gastrostomy (PEG) tubes have replaced nasogastric tubes and Stamm gastrostomy tubes as a means of feeding patients with head and neck carcinoma. PEG tubes are usually placed using the 'pull-technique,' or 'push technique' The pull technique, the first described method of PEG tube placement by Gauderer and Ponsky, is widely used [2]. In both of these techniques the tube is pulled or pushed through the oropharynx into position in the stomach by an endoscopically placed guide-wire which runs from the mouth to the stomach and through the anterior abdominal wall [3]. Since the introduction of this technique there have been at least fifteen case reports of tumour seeding at the gastrostomy site [4]. An alternative technique is the introducer technique (of Russell), which involves direct placement through the abdominal wall [5].

Surgical implantation of tumour cells is a well-recognized phenomenon that has been frequently reported in the literature. As early as 1885, Gertser put forward the view that implantation of wounds by malignant cells could be the cause of local recurrence. Lack in 1896 and Ryall in 1907 thought that 'infection' by malignant cells could occur and suggested that contaminated surgical equipment was responsible for implanting malignant cells in the operative field.

## Conclusion

Patients with head and neck carcinoma usually have gastrostomy tubes placed prior to surgery but where the feeding tube is passed over the oropharynx, tumour cells may be carried on the tube and directly implanted into the abdominal wall. In view of the fact that this is now at least the sixteenth report of port-site recurrence it may be more appropriate to insert a feeding gastrostomy tube laparoscopically at the time of the head and neck surgery. This simple minimally invasive technique overcomes the risk of implantation of cancer cells during insertion of a conventional PEG. I would recommend this technique for insertion of a feeding gastrostomy tube in patients with head and neck cancer.

## Conflict of interest

The author declare that he has no conflict of interest

## Authors' contributions

Single author paper
